# Antimicrobial Activity of the Essential Oil of *Plectranthus neochilus* against Cariogenic Bacteria

**DOI:** 10.1155/2015/102317

**Published:** 2015-06-16

**Authors:** Eduardo José Crevelin, Soraya Carolina Caixeta, Herbert Júnior Dias, Milton Groppo, Wilson Roberto Cunha, Carlos Henrique Gomes Martins, Antônio Eduardo Miller Crotti

**Affiliations:** ^1^Departamento de Química, Faculdade de Filosofia, Ciências e Letras de Ribeirão Preto, Universidade de São Paulo, 14040-901 Ribeirão Preto, SP, Brazil; ^2^Núcleo de Pesquisas em Ciências Exatas e Tecnológicas, Universidade de Franca, 14404-600 Franca, SP, Brazil; ^3^Departamento de Biologia, Faculdade de Filosofia, Ciências e Letras de Ribeirão Preto, Universidade de São Paulo, 14040-901 Ribeirão Preto, SP, Brazil

## Abstract

This work used the broth microdilution method to investigate the antimicrobial activity of the essential oil obtained from the leaves of *Plectranthus neochilus* (PN-EO) against a representative panel of oral pathogens. We assessed the antimicrobial activity of this oil in terms of the minimum inhibitory concentration (MIC). PN-EO displayed moderate activity against *Enterococcus faecalis* (MIC = 250 *μ*g/mL) and *Streptococcus salivarus* (MIC = 250 *μ*g/mL), significant activity against *Streptococcus sobrinus* (MIC = 62.5 *μ*g/mL), *Streptococcus sanguinis* (MIC = 62.5 *μ*g/mL), *Streptococcus mitis* (MIC = 31.25 *μ*g/mL), and *Lactobacillus casei* (MIC = 31.25 *μ*g/mL), and interesting activity against *Streptococcus mutans* (MIC = 3.9 *μ*g/mL). GC-FID and GC-MS helped to identify thirty-one compounds in PN-EO; *α*-pinene (**1**, 14.1%), *β*-pinene (**2**, 7.1%), *trans*-caryophyllene (**3**, 29.8%), and caryophyllene oxide (**4**, 12.8%) were the major chemical constituents of this essential oil. When tested alone, compounds **1**, **2**, **3**, and **4** were inactive (MIC > 4000 *μ*g/mL) against all the microorganisms. These results suggested that the essential oil extracted from the leaves of *Plectranthus neochilus* displays promising activity against most of the evaluated cariogenic bacteria, especially *S. mutans*.

## 1. Introduction

Dental caries is associated with acidogenic and aciduric bacteria that adhere to the tooth surface as an oral biofilm (dental plaque) [1]. Because this pathology can destroy dental hard tissues [2–4], it has become a major public health concern worldwide. The most efficient way to prevent caries and periodontal diseases is to reduce and eliminate bacterial accumulation on the top of and between teeth by brushing the teeth on a daily basis and conducting periodic dental cleaning or prophylaxis. Unfortunately, most people fail to maintain a sufficient level of oral hygiene [5], which has called for the use of oral products containing antimicrobial ingredients as a complementary measure to diminish biofilm formation on the tooth surface [6].

Chlorhexidine has been the most effective antiplaque agent tested to date, but some reversible local side effects have led dentists to recommend its use for short periods only [7]. Several other antimicrobial agents including fluorides, phenol derivatives, ampicillin, erythromycin, penicillin, tetracycline, and vancomycin can inhibit bacterial growth [8]. Nevertheless, excessive use of these chemicals can disturb the oral and intestinal flora and cause microorganism susceptibility, vomiting, diarrhea, and tooth staining [8]. To find an alternative to the substances currently employed to prevent caries and to control plaques, researchers have investigated the antimicrobial activities of natural products, especially essential oils [1, 3, 7, 9–11].

The herbaceous and aromatic plant* Plectranthus neochilus* is popularly known as “*boldo-rasteiro*” in Brazil [12]. In folk medicine, this plant has helped to treat disturbed digestion, skin infection, respiratory ailments [13], hepatic insufficiency, and dyspepsia [14]. The essential oil of* P. neochilus* displays antischistosomal [12] and insecticidal [15] activities. Recently, researchers have applied diffusion in agar disc to assess the antimicrobial activity of the essential oil of a specimen of* P. neochilus* collected in Portugal against* Bacillus cereus*,* Bacillus subtilis*,* Staphylococcus aureus*,* Listeria monocytogenes*,* Escherichia coli*,* Pseudomonas aeruginosa*,* Helicobacter pylori*, and* Saccharomyces cerevisiae* [16]. The authors reported that the activity of this essential oil against the selected microorganisms was between low and moderate.

As part of our ongoing research on the antimicrobial activities of essential oils [1, 17–19], in this work, we used the broth microdilution method to evaluate the* in vitro* antimicrobial activity of the essential oil obtained from the leaves of* Plectranthus neochilus* (Lamiaceae) against a representative panel of cariogenic bacteria.

## 2. Materials and Methods

### 2.1. Plant Material

Adult* P. neochilus* Schltr. (Lamiaceae) leaves were collected at “May 13th Farm” (20°26′S 47°27′W 977 m) in May 2011. The collection site was located near the city of Franca, state of São Paulo, Brazil. This species was identified by Professor Dr. Milton Groppo; one voucher specimen (SPFR 12323) was deposited at the Herbarium of the Department of Biology (Herbarium SPFR), University of São Paulo, Brazil.

### 2.2. Essential Oil Extraction

Fresh leaves of* P. neochilus* were submitted to hydrodistillation in a Clevenger-type apparatus for 3 h. To this end, 1200 g of the plant material was divided into three samples of 400 g each, and 500 mL of distilled water was added to each sample. Condensation of the steam followed by accumulation of the essential oil/water system in the graduated receiver of the apparatus separated the essential oil from the water, which allowed for further manual collection of the organic phase. Anhydrous sodium sulfate was used to remove traces of water. Samples were stored in an amber bottle and kept in the refrigerator at 4°C until analysis. Yields were calculated from the weight of the fresh leaves.

### 2.3. Gas Chromatography (GC-FID) Analyses

The essential oil of* P. neochilus* (PN-EO) was analyzed by gas chromatography (GC) on a Hewlett-Packard G1530A 6890 gas chromatograph fitted with FID and a data-handling processor. An HP-5 (Hewlett-Packard, Palo Alto, CA, USA) fused-silica capillary column (length = 30 m, i.d. = 0.25 mm, and film thickness = 0.33 *μ*m) was employed. The column temperature was programmed to rise from 60 to 240°C at 3°C/min and then held at 240°C for 5 min. The carrier gas was H_2_ at a flow rate of 1.0 mL/min. The equipment was set to the injection mode; the injection volume was 0.1 *μ*L (split ratio of 1 : 10). The injector and detector temperatures were 240 and 280°C, respectively. The relative concentrations of the components were obtained by peak area normalization (%). The relative areas were the average of triplicate GC-FID analyses.

### 2.4. Gas Chromatography-Mass Spectrometry (GC-MS) Analyses

GC-MS analyses were carried out on a Shimadzu QP2010 Plus (Shimadzu Corporation, Kyoto, Japan) system equipped with an AOC-20i autosampler. The column consisted of Rtx-5MS (Restek Co., Bellefonte, PA, USA) fused-silica capillary (length = 30 m, i.d. = 0.25 mm, and film thickness = 0.25 *μ*m). The electron ionization (EI-MS) mode at 70 eV was employed. Helium (99.999%) at a constant flow of 1.0 mL/min was the carrier gas. The injection volume was 0.1 *μ*L (split ratio of 1 : 10). The injector and the ion source temperatures were set at 240 and 280°C, respectively. The oven temperature program was the same as the one used for GC-FID. The mass spectra were registered with a scan interval of 0.5 s in the mass range of 40 to 600 Da.

### 2.5. Identification of the PN-EO Constituents

PN-EO components were identified on the basis of their retention indices relative to a homologous series of* n*-alkanes (C_8_–C_24_). To this end, an Rtx-5MS capillary column was employed under the same operating conditions as in the case of GC. The retention index (RI) of each PE-EO constituent was determined as described previously [20]. The chemical structures were computer-matched with the Wiley 7, NIST 08, and FFNSC 1.2 spectral libraries of the GC-MS data system; their fragmentation patterns were compared with the literature data [21].

### 2.6. Bacterial Strains and Antimicrobial Assays

The* in vitro* antimicrobial activity of PN-EO and its major constituents was assessed by minimum inhibitory concentration (MIC) values calculated by means of the broth microdilution method accomplished in 96-well microplates. The following standard ATCC strains were used:* Streptococcus salivarius* (ATCC 25975),* Streptococcus sanguinis* (ATCC 10556),* Streptococcus mitis* (ATCC 49456),* Streptococcus mutans* (ATCC 25175),* Streptococcus sobrinus* (ATCC 33478),* Enterococcus faecalis* (ATCC 4082), and* Lactobacillus casei* (ATCC 11578). Individual 24-h colonies from blood agar (Difco Labs, Detroit, MI, USA) were suspended in 10.0 mL of tryptic soy broth (Difco). Standardization of each microorganism suspension was carried out on a spectrophotometer (Femto, São Paulo, Brazil) operating at a wavelength (*λ*) of 625 nm, to match the transmittance of 81 (equivalent to 0.5 McFarland scale or 1.5 × 10^8^ CFU/mL). The microorganism suspension was diluted to a final concentration of 5 × 10^5^ CFU/mL. PN-EO was dissolved in DMSO (Merck, Darmstadt, Germany) at 16.0 mg/mL and diluted in tryptic soy broth (Difco), to yield concentrations between 4000 and 3.9 *μ*g/mL. Compounds** 1** (*α*-pinene),** 2** (*β*-pinene),** 3** (*trans*-caryophyllene), and** 4** (caryophyllene oxide) were purchased from Sigma-Aldrich (St. Louis, MA) and evaluated by means of the same methodology and at the same concentrations as PN-EO. A 1 *μ*M solution of each compound was tested individually. In the case of the mixture** 1** +** 2** +** 3** +** 4**, the constituents were mixed in the same proportion that they occurred in PN-EO. After dilutions, the DMSO concentrations were between 4% and 0.0039% (v/v). Three inoculated wells containing DMSO at concentrations ranging from 4% to 1% were used as negative controls. One inoculated well was included to control the adequacy of the broth for organism growth. One noninoculated well free of antimicrobial agent was also included to assess the medium sterility. Twofold serial dilutions of chlorhexidine dihydrochloride (CHD) (Sigma-Aldrich, St. Louis) were performed in tryptic soy broth (Difco) to achieve concentrations ranging from 5.9 to 0.115 *μ*g/mL. These dilutions were used as positive control. The microplates (96-well) were sealed with parafilm and incubated at 37°C for 24 h. After that, 30 mL of 0.02% resazurin (Sigma-Aldrich, St. Louis, MO, USA) aqueous solution was poured into each microplate reservoir to indicate microorganism viability [22]. The MIC value (i.e., the lowest concentration of a sample capable of inhibiting microorganism growth) was determined as the lowest concentration of the essential oil and or major constituents capable of preventing a colour change of the resazurin solution [23]. Three replicates were conducted for each microorganism.

## 3. Results and Discussion

This work relied on minimum inhibitory concentration (MIC) values to evaluate the antimicrobial activity of the essential oil of* P. neochilus* (PN-EO) against a panel of cariogenic bacteria; chlorhexidine dihydrochloride (CHD) was the positive control. Samples with MIC values lower than 100 *μ*g/mL, between 100 and 500 *μ*g/mL, and between 500 and 1000 *μ*g/mL were considered to be promising, moderately active, and weak antimicrobials, respectively. Samples with MIC values greater than 1000 *μ*g/mL were deemed inactive [11, 24–26].

Table 1 summarizes the MIC values. PN-EO displayed moderate activity against* S. salivarius* (MIC = 250 *μ*g/mL) and* S. faecalis* (MIC = 250 *μ*g/mL) and significant antimicrobial activity against* Streptococcus sobrinus* (MIC = 62.5 *μ*g/mL),* Streptococcus sanguinis* (MIC = 62.5 *μ*g/mL),* Streptococcus mitis* (MIC = 31.25 *μ*g/mL),* Lactobacillus casei* (MIC = 31.25 *μ*g/mL), and* Streptococcus mutans* (MIC = 3.9 *μ*g/mL). The antimicrobial activity of PN-EO against* S. mutans* was an interesting result: this microorganism is considered to be the main cariogenic agent [10, 27], and very few natural compounds can inhibit it [26].

Hydrodistillation of* P. neochilus* leaves afforded PN-EO in 0.03% ± 0.01 (w/w) yield. Gas chromatography revealed the presence of 31 compounds in PN-EO, namely, fifteen monoterpenes (36.0%), fifteen sesquiterpenes (63.5%), and aliphatic alcohol (0.2%). The major PN-EO constituents were *α*-pinene (**1**; 14.1%), *β*-pinene (**2**; 7.1%),* trans*-caryophyllene (**3**; 29.8%), and caryophyllene oxide (**4**; 12.8%), as shown in Table 2. The chemical composition of PN-EO differed significantly from the chemical composition of* P. neochilus* specimens collected in South Africa, whose major constituents were citronellol (29.0%), citronellyl formate (11.0%), linalool (9.8%), and isomenthone (9.2%) [28], but it resembled the chemical composition previously reported for* P. neochilus* specimens collected in Brazil [12, 15]. These different chemical compositions may be associated with environmental factors or growing conditions, which can greatly affect the chemical composition of volatile oils [29, 30].

The antimicrobial activity of essential oils has been associated with the lipophilicity of their chemical constituents, mainly monoterpenes and sesquiterpenes, which are often the main chemicals thereof [31]. The hydrophobicity of terpenoids would allow these compounds to diffuse across the cell membranes easily and to kill microorganisms by affecting the metabolic pathways or organelles of the pathogen. In addition, synergistic interactions between essential oil components could enhance their activity [31]. For this reason, the major chemical constituents of some essential oils deserve antimicrobial evaluation alone or as a mixture [32–34]. This study evaluated the individual antimicrobial activity of *α*-pinene (**1**), *β*-pinene (**2**),* trans*-caryophyllene (**3**), and caryophyllene oxide (**4**). Alone, all of these compounds were much less effective against the selected cariogenic bacteria than PN-EO; their MIC values were higher than 4000 *μ*g/mL (Table 1). The antimicrobial activity of a mixture containing compounds** 1** +** 2** +** 3** +** 4** in the same relative proportion compared to their relative areas in the CG-FID chromatogram of PN-EO displayed moderate activity against* L. casei* (MIC = 500 *μ*g/mL) and weak activity against* S. mutans* (MIC = 1000 *μ*g/mL), but it was inactive against the other bacteria (MIC > 4000 *μ*g/mL). Although the MIC values obtained for the mixture suggested a very discrete synergism between compounds** 1**,** 2**,** 3**, and** 4**, the mixture** 1** +** 2** +** 3** +** 4** was much less active than PN-EO. Hence, only the presence of compounds** 1**,** 2**,** 3**, and** 4** does not account for the antimicrobial activity of PN-EO. In fact, the antimicrobial activity of PN-EO may also be related to the other minor chemical constituents identified in the oil, which may underlie or even increase the activity of the major chemical constituents of this essential oil.

## 4. Conclusions

The essential oil of* P. neochilus* (PN-EO) displays promising antimicrobial activity against some cariogenic bacteria, including* Streptococcus mutans*, which is one of the main causative agents of dental caries. Taken together, our results suggest that this essential oil might be promising for the development of new oral care products. Further studies to identify the active chemical constituents of PN-EO are underway.

## Figures and Tables

**Table 1:** Minimum inhibitory concentration (MIC) values (*μ*g/mL) obtained for the essential oil of *P. neochilus* (PN-EO), compounds **1**, **2**, **3**, and **4**, and the mixture **1** + **2** + **3** + **4** against selected cariogenic bacteria.

Microorganisms	PN-EO	CHD^a^	**1** ^b^	**2** ^b^	**3** ^b^	**4** ^b^	**1** + **2** + **3** + **4** ^c^
*E. faecalis *	250.0	14.8	>4000	>4000	>4000	>4000	>4000
*S. salivarius *	250.0	7.4	4000	4000	4000	4000	4000
*S. mutans *	3.9	1.8	>4000	>4000	>4000	>4000	1000
*S. mitis *	31.3	14.8	4000	4000	4000	4000	4000
*S. sobrinus *	62.5	1.8	>4000	>4000	>4000	>4000	4000
*S. sanguinis *	62.5	7.4	4000	>4000	>4000	>4000	>4000
*L. casei *	31.3	3.7	4000	4000	4000	4000	500

^a^Chlorhexidine dihydrochloride.

^b^For compounds **1**, **2**, **3**, and **4**, the concentration of 4000 *μ*g/mL corresponds to 29.4, 29.4, 19.6, and 18.1 mM, respectively.

^c^ **1** + **2** + **3** + **4**: mixture of *α*-pinene, *β*-pinene, *trans*-caryophyllene, and caryophyllene oxide.

**Table 2:** Chemical composition of the essential oil from the leaves of *P. neochilus* as identified by GC/MS.

Chemical compound	RT [min]^a^	RI_exp⁡_^b^	RI_lit_^c^	Content [%]^d^	Identification^e^
*α*-Thujene	5.00	921	924	6.3	RL MS
*α*-Pinene (**1**)	5.19	929	932	14.1	RL MS
Thuja-2,4(10)-diene	5.43	939	941	0.2	RL MS
Camphene	5.62	943	947	0.1	RL MS
Sabinene	6.21	966	971	1.9	RL MS
*β*-Pinene (**2**)	6.37	975	977	7.1	RL MS
*β*-Myrcene	6.65	985	988	0.3	RL MS
Octan-3-ol	6.90	993	996	0.2	RL MS
*α*-Terpinene	7.53	1015	1016	0.5	RL MS
o-Cymene	7.79	1022	1023	0.3	RL MS
Limonene	7.94	1026	1027	0.2	RL MS
(*Z*)-*β*-Ocimene	8.14	1030	1033	0.4	RL MS
(*E*)-*β*-Ocimene	8.50	1040	1043	1.8	RL MS
*γ*-Terpinene	8.94	1052	1055	1.4	RL MS
*α*-Terpinolene	9.94	1080	1084	0.2	RL MS
4-Terpineol	13.75	1177	1179	1.2	RL MS
*α*-Cubebene	20.78	1341	1344	0.5	RL MS
*α*-Copaene	21.97	1366	1372	1.2	RL MS
*β*-Bourbonene	22.29	1380	1379	1.1	RL MS
*β*-Cubenene	22.50	1378	1384	0.3	RL MS
*trans*-Caryophyllene (**3**)	23.80	1412	1415	29.8	RL MS
*α*-Humulene	25.25	1448	1450	1.5	RL MS
Germacrene D	26.30	1470	1476	6.2	RL MS
Eremophilene	27.51	1504	1505	3.9	RL MS
*α*-Amorphene	27.61	1506	1508	0.4	RL MS
*δ*-Cadinene	27.83	1514	1513	1.9	RL MS
(*E*)-Nerolidol	29.63	1554	1559	0.3	RL MS
Caryophyllene oxide (**4**)	30.26	1571	1575	12.8	RL MS
Unknown	32.06	—	1622	0.3	—
*epi*-*α*-Cadinol	32.62	1634	1637	1.5	RL MS
*δ*-Cadinol	32.70	1636	1639	0.8	RL MS
*α*-Cadinol	33.12	1647	1650	1.3	RL MS

Monoterpenes hydrocarbons				34.8	
Oxygenated monoterpenes				1.2	
Sesquiterpenes hydrocarbons				46.8	
Oxygenated sesquiterpenes				16.7	
Others				0.2	
Not identified				0.3	

^a^RT: retention time determined on the Rtx-5MS capillary column.

^b^RI_exp⁡_: retention index determined on the Rtx-5MS column relative to *n*-alkanes (C_8_–C_20_).

^c^RI_lit_: retention index.

^d^Calculated from the peak area relative to the total peak area.

^e^RL: comparison of the retention index with the literature [21]; MS: comparison of the mass spectrum with the literature.
